# Contrast-enhanced near-infrared photoacoustic microscopy and optical coherence tomography imaging of rat fundus

**DOI:** 10.1515/nanoph-2023-0872

**Published:** 2024-06-13

**Authors:** Fengxian Du, Chen Niu, Silue Zeng, Jingqin Chen, Chengbo Liu, Cuixia Dai

**Affiliations:** 92263 Shanghai Institute of Technology, Shanghai 201418, China; Shenzhen Institute of Advance Technology Chinese Academy of Sciences, Shenzhen 518055, China

**Keywords:** optical coherence tomography, photoacoustic microscopy, contrast agents

## Abstract

In this paper, we design a multimodal visible/near-infrared photoacoustic microscopy and optical coherence tomography (VIS/NIR-PAM-OCT) system for imaging both retina and retinal pigment epithelium (RPE)/choroid complex layer. F127 and DSPE-PEG-cRGD encapsulated IR-1048 nanoparticles (FINPs) exhibiting absorption peak up to 1,064 nm were utilized as contrast agents to enhance NIR-PAM for *in vivo* imaging of fundus tissues. The fundus structure and vessels are clearly visualized by the multimodal imaging, and their parameters were quantitatively analyzed. NIR-PAM and OCT imaging of fundus were time-serially monitored over 60 min following the intravenous injection of FINPs into rats. The results indicated a 134 % increase in image signals in PAM at 1 min, along with an 8.23 % intensity enhancement in OCT. Moreover, laser-induced choroidal neovascularization (CNV) was specifically detected and accurately quantified using VIS/NIR-PAM-OCT. Lastly, FINPs demonstrated excellent biocompatibility in hematology analysis and pathology testing.

## 1 Introduction

The association between fundus abnormality and various ocular disorders, such as high myopia, age-related macular degeneration (AMD), choroidal tumors, and posterior uveitis, has been identified [1], [2], [3], [4], [5]. Visualizing the fundus is essential for clinical diagnosis and ophthalmic research. Currently, fluorescein fundus angiography (FFA) is used as a traditional detection technique for fundus disease [6], and fundus photography is regarded as the gold standard for disease screening [7]. Nevertheless, it is important to note that both FFA and fundus photography are restricted to capturing two-dimensional images of the fundus, lacking the ability to distinguish overlapping fundus information. Alternatively, ultrasound imaging (UI) and magnetic resonance imaging (MRI) have the advantages of 3D imaging and long imaging depth which can image the entire fundus, but the resolution is not high enough to differentiate the retinal and choroidal layers [8], [9]. In recent years, optical coherence tomography (OCT) and photoacoustic imaging (PAI) are highly recommended for fundus detection due to their advantages of non-invasive cross-sectional imaging [10], [11], [12]. Simultaneous PAI and OCT imaging will provide sensitive and specific features of fundus tissues, demonstrating both optical absorption and scattering contrasts.

In recent research, Zhang and co-authors firstly applied an OCT-guided visible photoacoustic microscopy (VIS-PAM) system to image fundus diseases in albino rats [13]. Jiao and co-authors presented an 800 nm PAM and OCT system for fundus imaging in pigmented rats and found deeper detection of PAM using longer wavelength [14]. Subsequently, Paulus and co-authors successfully achieved the 700 nm PAM and OCT imaging for choroidal neovascularization (CNV) detection in albino rabbits [15]. In our recent study, the VIS-PAM and OCT system were combined to detect laser-induced retinal neovascularization (RNV) and CNV in rats to provide sensitive and specific features [16], [17].

In recent few years, detecting tissues below the retina was highly desired. Light sources with longer wavelengths (>800 nm) are preferable for extended depth imaging due to their increased ability to penetrate deeper tissues. In OCT imaging, 1.0 μm wavelength light source is successfully used for both retina and choroid detection. In PAM imaging, the typical use of short wavelength light sources (532 nm–800 nm) enables the visualization of retina vessels, but it is unable to image the RPE/choroid complex layer [13], [14], [18]. Therefore, it is concerned to utilize a wavelength range of 1,000–1,700 nm in the near-infrared bands (NIR-II) for PAM imaging [19], [20]. Due to the advantages of larger penetration depth and higher maximum permissible exposure of incident light, PAM in the second near-infrared window is more suitable for imaging the RPE/choroid complex layer. And the advantage of NIR-II light in ophthalmic imaging, which is safer and more comfortable for eyes, has been generally accepted in clinical practice.

However, the other side of the coin of using near-infrared light source in PAM is that the image contrast is limited by the weak absorption of blood cells. To address the issue of inadequate contrast imaging, contrast agents are consistently employed to augment the imaging sensitivity. In recent years, combined with nanoparticles as exogenous contrast agents, NIR-photoacoustic computed tomography (PACT) imaging enabled the visualization of biological tissue at depths of several centimeters *in vivo* [21].

Current NIR-II probes range from inorganic materials, small-molecule dyes, to conjugated polymer-based materials [22], [23], [24]. The safety concerns surrounding the long-term use of inorganic materials have limited their development. Small-molecule dyes display strong NIR-II absorption and good biocompatibility but often suffer from poor photostability. Polymer probes begin to emerge as promising candidates for PA based diagnosis owing to both strong NIR-II absorption and scattering, high photostability and good biocompatibility. In this paper, to visualizing the fundus vessels at NIR-II illumination, we synthesized a polymer nanoparticle as exogenous contrast agent. FINPs were used as a brilliant photoacoustic dual-modal contrast agent, which were proved to have strong effect to image deep biological tissues at NIR-II bands. To the best of our knowledge, this study is the first time to utilize FINPs to improve multimodal NIR-PAM and OCT imaging of the rat fundus.

In this study, based on our previous research [16], [17], we developed the integration of VIS-PAM (532 nm light source), NIR-PAM (1,064 nm light source) with SD-OCT (central wavelength: 840 nm) for multimodal imaging system (VIS/NIR-PAM-OCT) of both optical absorption and scattering contrasts of the retina and RPE/choroid complex layer in normal rats and CNV model rats. The retina and the RPE/choroid complex layers were simultaneously visualized. Polyethylene glycol modified FINPs with absorption peaks up to 1,064 nm were synthesized as a contrast agent to improve the image contrast and signal intensity. Since excellent nanoparticles lead to saturation of the image signal, the photoacoustic signal can be acquired by reducing the laser pulse energy. The use of low-energy pulse signals enhances the safety of eye imaging.

## 2 Methods and materials

### 2.1 Experimental materials

**Figure 1:**
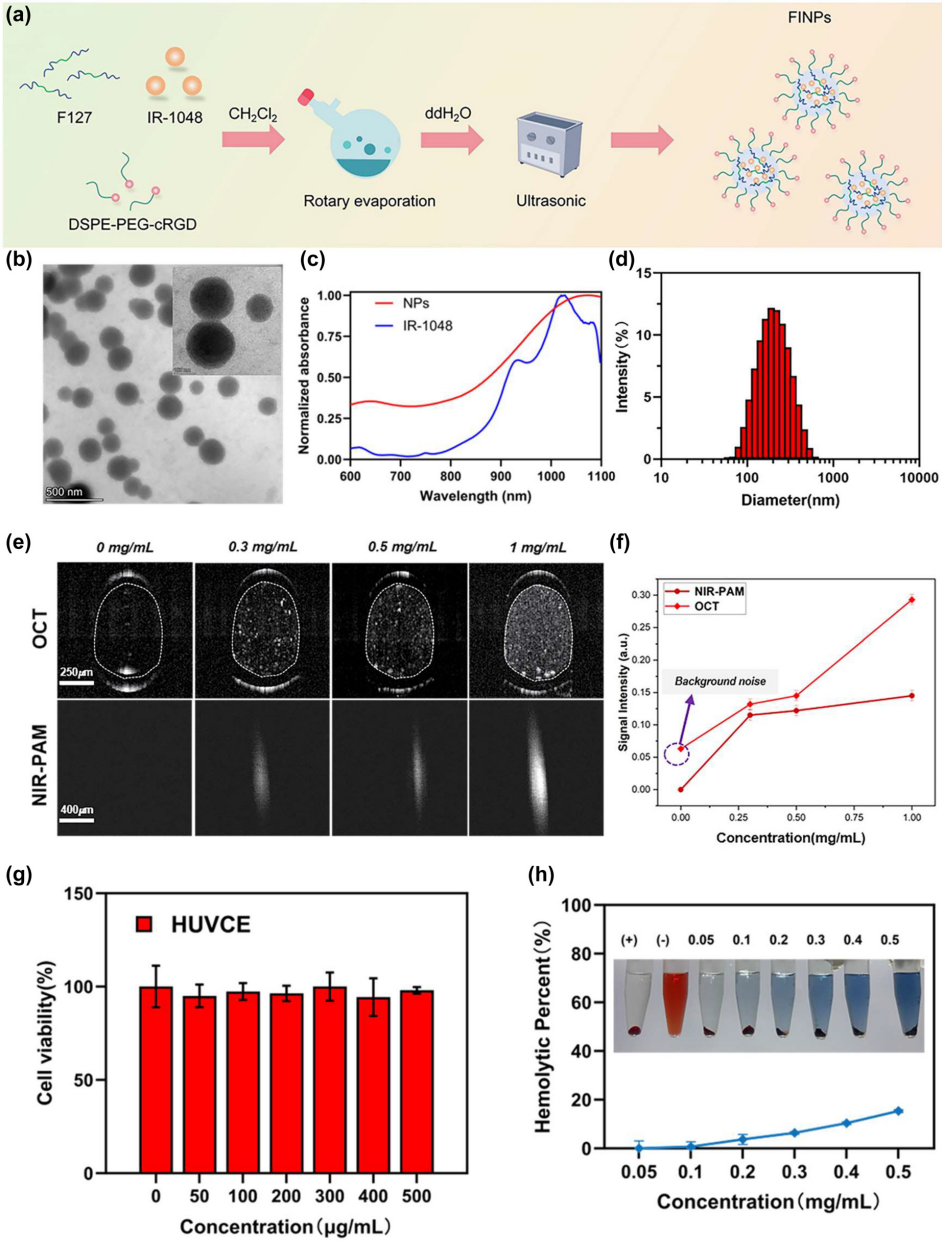
Characterization of FINPs, optical properties, and biocompatible analysis. (a) Illustration of synthesis of FINPs. (b) Transmission electron microscopy (TEM) image of FINPs, bar = 500 nm. (c) Normalized VIS-NIR absorption spectra of FINPs solution and the IR-1048 in CHCl_3_ solution. (d) DLS measurement of TCZ-PNPs in aqueous solution. (e) OCT and PAM images of phantoms at 0.3 mg/mL, 0.5 mg/mL, 1.0 mg/mL FINPs and saline, respectively. (f) OCT and PAM signal are detected at saline and 0–1.0 mg/mL FINPs and OCT and PAM signal amplitudes are quantitative analysis. (g) Viabilities HUVCE incubated with FINPs at different concentrations for 24 h and HUVCE viability higher than 85 % prove that the nanoparticles had low cytotoxicity. (h) Hemolytic percentage of red blood cells after treatment with FINPs for 3 h at various concentrations, using deionized water (+) and phosphate buffer saline (−) as positive and negative controls, respectively. The inset show the photographs for direct observation of hemolysis.

As shown in Figure 1A, to fabricate the NIR-II-absorbing nanoparticles, the NIR-II small molecule dye IR-1048 was used as an absorber. Pluronic F127 was added to the composite for improved dispersibility and solubility. Polyethylene glycol (PEG) was adopted to increase the circulation of nanoparticles in the bloodstream and to enhance permeability and retention effect (EPR). We employed cyclic RGD peptides (cRGD) to specifically target CNV. In brief, IR-1048, F127 and DSPE-PEG-cRGD (mass ratio = 1:50:10) were mixed and dissolved in dichloromethane, and the mixture was then placed in an ultrasonic processor (SONICS, VCX150, 150 Hz, 35 %) for 30 min. Afterwards, the mixture was evaporated with a rotary evaporator to remove the dichloromethane, giving a thin film. Next, it was dispersed by DI water with 30 min ultrasonication, resulting in the final 1 mg/mL FINPs solution.

To characterize the optical properties of FIPNs, they are examined morphologically by transmission electron microscopy (TEM), which shows that FINPs is uniform spherical (Figure 1B). The average size of FINPs is determined to be 180 nm by dynamic light scattering (DLS) analysis as shown in Figure 1D. The near infrared absorption band of the FINPs is observed at a wavelength of 1,064 nm, which closely resembles the absorption peak of the IR-1048 dye in a dichloromethane solution (Figure 1C).

To verify the scattering properties of nanoparticles, a group of capillary glass tubes (inner: 0.3 mm, outer: 0.5 mm) filled with FINPs with different concentrations (0.3, 0.5 and 1 mg/mL) and the control group filled with saline were imaged by OCT system. As shown in Figure 1E, in which intensity value in the white dotted ellipse in OCT B-scan images were averaged. The OCT scattering signal of FINPs at concentrations 1 mg/mL exhibited 3.5-fold higher than that induced by saline (Figure 1F). In addition, polystyrene tube was chosen to verify the absorption properties of the nanoparticles because it has a relatively low acoustic impedance and acoustic attenuation. Polystyrene tubes (inner: 0.28 mm, outer: 0.64 mm) filled with FINPs with different concentrations (0.3, 0.5 and 1 mg/mL) and the control group filled with saline were imaged by PAM system. We utilized the same single polystyrene tube to acquire the PA signal intensity with contrast agents at different concentrations. The pixel values of each PAM *en face* image was averaged and then normalized. Signal intensity of PAM at 1.0 mg/mL of FINPs was 144.6 % high of that using saline (Figure 1F). Thus, a concentration of 1.0 mg/mL was selected as an exogenous contrast agent for intravenous injection *in vivo*.

The standard Cell Counting Kit-8 assay and hemolytic analysis were conducted to evaluate the cytotoxicity of nanoparticles on cells and hemocompatibility. Cell viability was measured after 24 h incubation of human umbilical venous cord endothelial cell (HUVCE) with different concentrations FINPs. Obviously, HUVCE viability higher than 85 % proved that the nanoparticles had low cytotoxicity, as shown in Figure 1G. In addition, different concentrations of FINPs (0.05, 0.1, 0.2, 0.3, 0.4, and 0.5 mg/mL) were determined for hemolytic effects with red blood cells, which was used to evaluate the hemocompatibility. Positive and negative controls were deionized water and PBS, respectively. As we can see in Figure 1H, FINPs were compatible with blood. Thus, FINPs synthetized for this study have good biocompatibility. FINPs exhibit excellent PAM absorptive effects and great OCT scattering effects. A variety of high-quality features show that FINPs have great potential for PAM-OCT imaging *in vivo* [21], [25].

### 2.2 Experimental setup

**Figure 2:**
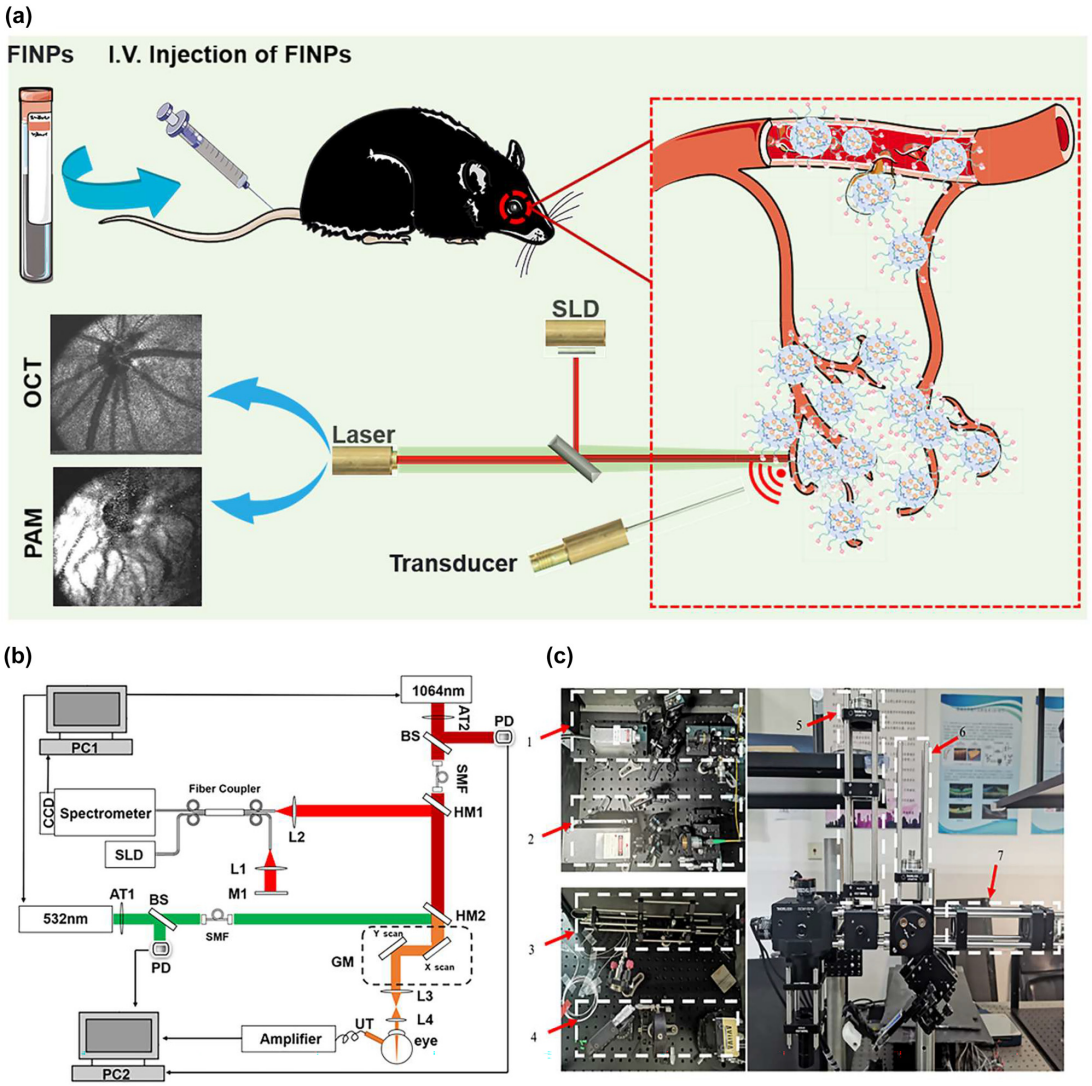
Experimental setup of multimodal VIS/NIR-PAM-OCT system. (a) Illustration of the *in vivo* multimodal imaging using FINPs. The synthesized FINPs were intravenously injected into the rat model through vein. OCT and PAM signals from the rat fundus was generated by using superluminescent diodes (SLD) and nanosecond pulsed laser illumination at wavelength of either 532 or 1,064 nm. (b) Schematic diagram of the dual-modal imaging system. PC1, PC2, personal computer for scanning controlling and data acquisition; SLD, superluminescent diode; AT1, AT2, adjustable attenuator; BS, beam splitter; PD, photodiode; SMF, single mode fiber; HM1, HM2, hot mirror; GM, two-dimensional galvanometer; UT, ultrasonic transducer; M1, mirror; L1-L4, lens. (c) Physical setup of the dual-modal imaging system. 1,2-fiber coupled. 3-reference arm. 4-homebuilt spectrometer. 5,6,7-beam alignment.

A multimodal imaging system of dual-wavelength PAM (VIS-PAM and NIR-PAM) and spectral domain OCT (SD-OCT) was set up, as shown in Figure 2A–C.

In the PAM system, near-infrared semiconductor lasers (MPL-H-1064; output wavelength: 1,064 nm; pulse energy: 19 μJ; pulse duration: 5 ns; pulse repetition rate: 5 kHz; Changchun New Industries Co., Ltd) and visible light semiconductor laser (MPL-H-532, output wavelength: 532 nm; pulse energy: 15 μJ; pulse duration: 4 ns; pulse repetition rate: 5 kHz; Changchun New Industries Co., Ltd) were used as the illumination sources for PAM imaging. To adhere to laser safety thresholds, it was necessary to attenuate (NDC-25C-4M, Thorlabs) the output power of the laser. The 1,064 nm and 532 nm light were coupled into two single-mode optical fibers. A dual-axis galvo system (GVS002, Thorlabs) scanned the laser beam and delivered the light onto the fundus using a relay lens (AC254-075-AB, focal length: 75 mm, Thorlabs) and an objective lens (AC127-019-AB, focal length: 19 mm, Thorlabs). Photoacoustic signals induced by the optical absorption of hemoglobin and melanin were captured by a custom-built unfocused needle ultrasonic transducer (40-MHz central frequency; 0.5 mm × 0.5 mm active element size).

The transducer was placed in contact with the eyelid coupled using a drop of ultrasound gel for signal coupling. The PAM signal was amplified by two amplifiers (ZFL-500LN-BNC+, 0.1–500 MHz, Mini-circuits), and was digitized by a data acquisition board (CSE161G2, Gage) at a sampling rate of 1.00 GS/s. Respectively, the visible laser pulse energy was 60 nJ and the near-infrared laser pulse energy was 450 nJ, which was reported to be eye safe by different groups [26], [27], [28]. For the SD-OCT system, the near infrared light (IPSDD0804, InPhenix, CA; central wavelength: 840 nm; 6-dB bandwidth: 50 nm) was split to reference arm and sample arm by a 50 × 50 customized single-mode fiber optic coupler, and the sample arm was coupled with the NIR-PAM by a hot mirror (69–902, Edmund Optics) with the illumination power 0.8 mW. A homebuilt spectrometer consisted of a grating (Wasatch, 1,800 lines/mm), a focusing lens (AC508-100-B, focal length:100 mm, Thorlabs), and a line camera (Aviiva SM2, e2v) was used. The PAM and OCT systems were coaxially aligned to ensure coregistration of the dual-modality images. As shown in Figure S1 A, the laser irradiation and distribution during fundus imaging are simulated using Zemax software based on the structural parameters of the rat eye, the lateral imaging resolution is calculated to be ∼20 µm. In Figure S1 B–C, the longitudinal resolution of PAM and SD-OCT are quantified to be 35 μm and 6.4 μm, respectively. The imaging depth of the OCT system is tested to be 3.6 mm. Figure S1 D exhibits an OCT B-scan image of layered Scotch tape with ten distinct layers being identifiable.

### 2.3 Animals preparation

In this study, normal BN rats (7–8 weeks, weight: 160 g) were provided by Shanghai Laboratory Animal Research Center (Shanghai, China). Laser-induced rupture of Bruch’s membrane in BN rats (7–8 weeks, weight: 160 g) was used as the CNV model rats. During experiments, rats were anesthetized with 1 % Pentobarbital sodium solution, which was injected intraperitoneally into the animals with a dosing of 40 mg/kg body weight. Anesthetized animals were restrained in a home-built holder, which was then placed on an adjustable platform with five degrees of freedom. Before imaging, we dilated the rodents’ pupils with a 1 % tropicamide ophthalmic solution and paralyzed the iris sphincter muscle with a 0.5 % tetracaine hydrochloride ophthalmic solution. Artificial teardrops (Systane, Alcon Laboratories, Inc.) were applied every other minute to prevent corneal dehydration. The rat’s body temperature needed to be monitored during intravenous injection. All animals were kept in a pathogen free environment and fed ad-lib. The procedures for care and use of animals were approved by the Ethics Committee of the Shenzhen Institute of Advanced Technology Chinese Academy of Sciences and all applicable institutional and governmental regulations concerning the ethical use of animals were followed.

## 3 Results

### 3.1 VIS/NIR-PAM-OCT imaging and quantitative analysis of fundus vessels in normal rats

**Figure 3:**
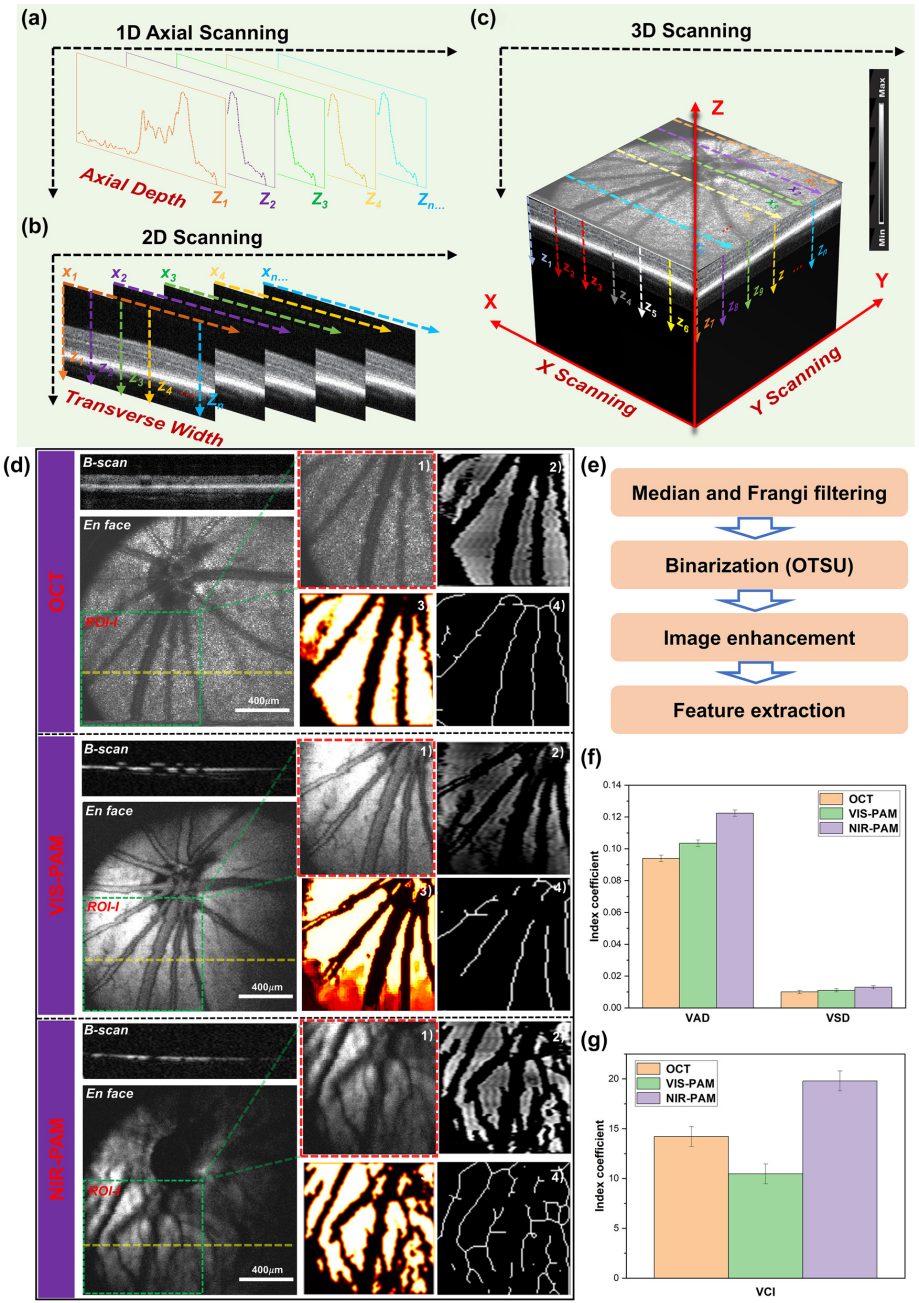
Multimodal photoacoustic microscopy and optical coherence tomography imaging of rat fundus. (a)–(c) A schematic diagram of 3D reconstructions of the signal. (d) OCT, VIS-PAM, and NIR-PAM *en face* and B-scan image of rat fundus without nanoparticle injection. The dotted yellow line indicates the corresponding B-scan image. *En face* image of ROI-I (64 × 64 pixels) selected from original image (256 × 256 pixels) are shown as green rectangles. The quantitative analysis of vasculature in ROI-I. (1) The original image. (2) The vessel image acquired using hessian filter and threshold segmentation. (3) Blood vessel denoising and enhancement. (4) The vessel vasculature skeleton map. (e) The flowchart of the vascular extraction algorithm. (f–g) The quantitative analysis of VAD, VSD, and VCI vascular parameters.

Before injecting nanomaterials, the rat fundus was imaged with VIS/NIR-PAM-OCT system to detect PAM and OCT signals. In simultaneous OCT and PAM imaging, we performed 256 A-lines in each B-scan and scanned 256 discrete B-scan positions to produce a volumetric map of the fundus images, as shown in Figure 3A–C. The final image volume of data is 256 × 256 × 2048 pixels, as shown in Figure 2C (*x*-256, *y*-256, *z*-2048). Both OCT and PAM had the same B-scan frame rate, which was 19.5 frame/s. And it took 13 s to acquire the entire image volume. OCT *en face* images were generated from the 3D OCT dataset, and intensity projection was performed in the depth direction to obtain the summation of the pixel intensity. PAM *en face* images were produced from the maximum amplitude projection (MAP) of the dataset. After the fundus area of interest was accurately positioned by OCT, PAM was used to perform specific vascular imaging of the area. The combination of the two imaging techniques enabled us to obtain the optimal of structural information and specific blood vessels of the fundus.

For healthy fundus of normal rat, OCT and PAM *en face* images provide typical scattering and absorbing characters of the same location of fundus, respectively. As shown in Figure 3D, the OCT *en face* images provide a typical retina fundus. The OCT B-scan image presents high reflection from the inner layers and a weak reflection from the outer layers. The retinal pigment epithelium (RPE) and choriocapillaris are observed as bands of high reflection. In the VIS-PAM *en face* images depicted in Figure 3D, it is observed that the choroid vessels cannot be well resolved due to the limited penetration depth of the visible light. For NIR-PAM images, weak NIR-PAM signal was detected using near infrared light due to low optical absorption of melanin and hemoglobin at 1,064 nm wavelength. Compared with VIS-PAM images, the near-infrared pulsed laser can penetrate the retinal layer and cause the melanin around the deep choroidal vessels to absorb the laser energy to generate more detailed vascular information in NIR-PAM images. Correspondingly, VIS-PAM B-scan images provide high absorption contrast of retina vessels and RPE layer and NIR-PAM cross-sectional images only shown the low absorption properties of the melanin.

For NIR-PAM images, the shadowed vessels were deeply analyzed to be not only choroidal vessels but also some small retinal vessels, as demonstrated in Supplementary Figure S2. The representative 220th B-scan image was extracted from both the OCT and NIR-PAM datasets utilizing the original data. A comparison with *en face* images from OCT and NIR-PAM reveals that the vessels at positions line 3, line 4, and line 5 correspond to larger retinal vessels. For a more intuitive display of the two-dimensional data in the B-scan images, we employed contour mapping to delineate the pixel value trends in the fundus images, which distinctly reflect regions of significant pixel gradients. The deeper colored elliptical areas in the OCT B-scan images correspond to these larger retinal vessels, which are also conspicuously highlighted in the contour maps derived from the OCT B-scans. Notably, the bright layer in the OCT B-scan images is indicative of the high-scattering signal from the RPE. In the corresponding NIR-PAM B-scan images, the retinal vessels cast shadows due to their obstruction of the RPE layer, resulting in variable contrast. In Figure S2, there is no visibility in the OCT *en face* or the VIS-PAM *en face* images for vessels at positions line 1. They only appear in NIR-PAM images. The OCT B-scan images exhibit elliptical background colors reminiscent of those observed at places line 3, 4, and 5. The contour maps clearly demonstrate the presence of a highlighted backdrop beneath the RPE layer, as well as partial deformation of the RPE layer. The NIR-PAM B-scan images exhibit a lack of continuity in the RPE/choroid complex layer signals due to the presence of vessels. We interpret this phenomenon as that the photoacoustic signal in NIR-PAM primarily originating from melanin within the RPE and choroidal layers [29], [30]. The vessels present within the choroidal layer create a differential contrast in photoacoustic signals due to their location relative to surrounding melanin. Therefore, it is believed that the features observed at line 1 correspond to choroidal blood vessels. Regarding the vessel at line 2, located above the RPE layer in the OCT B-scan, its presence causes discontinuity in the NIR-PAM B-scan signal – a characteristic indicative smaller retinal vessels. Following the application of signal averaging reconstruction algorithms, OCT *en face* images made smaller retinal vessels less distinct due to the higher scattering signals. In the VIS-PAM *en face* images, the maximum projection algorithm fails to reveal these vessels because the absorption of visible light by melanin in the RPE layer vastly overwhelms the signal from smaller retinal vessels, effectively obliterating their shadow signals and thus their contrast.

To conduct a thorough assessment of the ability of NIR-PAM to acquire more intricate vessel data, the same region of interest (ROI-I) of the fundus vessel image (64 × 64 pixels) were selected. The representative morphological parameters, vessel area density (VAD), vessel skeleton density (VSD), and vessel complexity index (VCI) were selected to quantify blood vessel information [31], [32], [33], [34], [35]. The specific processing flow was shown in Figure 3E. The original image was converted to a binary image using MATLAB (R2022a, MathWorks, Inc.) based on a hessian filter and an adaptive threshold. This was an effective approach to achieve the preliminary segmentation of detailed vessel information, and important vessel information was illustrated in the binary image. Then a vessel skeleton map was established, where all the vessels are represented as pixel lines, regardless of their diameters. The length information of vessel was extracted via skeletonization. Finally, the abovementioned vessel information parameters were calculated based on these vessel maps. In Figure 3F, it can be observed that both VAD and VSD achieve higher precision under our NIR-PAM. As for VCI, the complexity of vessels obtained by NIR-PAM is significantly higher than that obtained by OCT and VIS-PAM as shown in Figure 3G. Therefore, our results quantitatively demonstrate the significance of NIR-PAM for fundus vessel *in vivo* imaging.

However, although the detailed information of blood vessels can be quantified by NIR-PAM, the overall signal-to-noise ratio (SNR) of the image is poor. Low-signal NIR-PAM images lead to the loss of a lot of blood vessel information. The overall average signal of NIR-PAM *en face* images is lower than that of OCT and VIS-PAM images. Moreover, vessels on the right half of the images that deviate from the needle ultrasound transducer are almost indistinguishable. Therefore, it is important to further improve the vascular information of NIR-PAM images *in vivo*.

### 3.2 FINPs enhanced NIR-PAM and OCT imaging of fundus in normal rats

**Figure 4:**
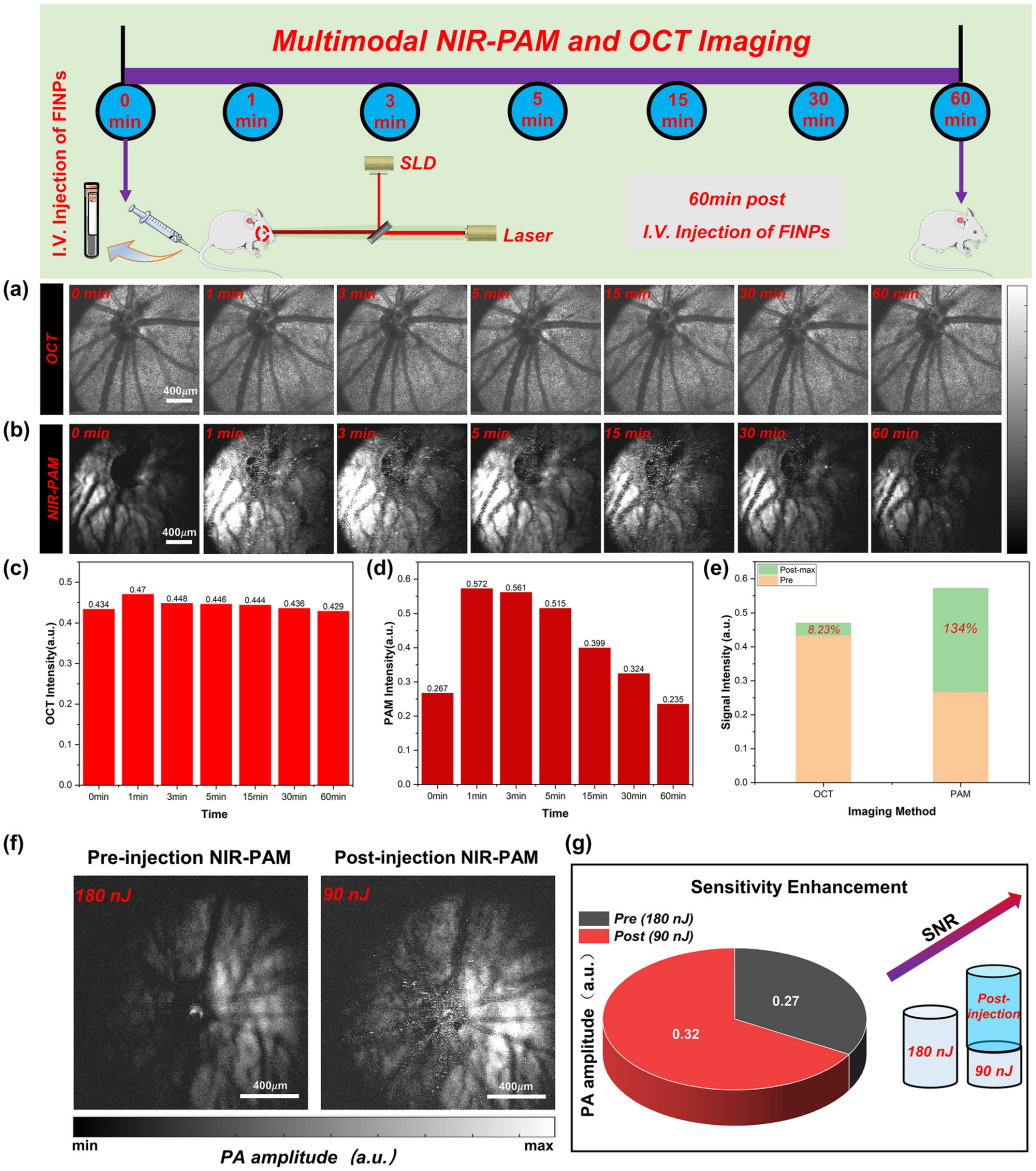
Normal rat fundus was time-serially monitored and quantified analysis of signals. (a) Normal rat fundus was time-serially monitored with OCT. (b) Normal rat fundus was time-serially monitored with NIR-PAM. (c) OCT signal intensity was detected at 1–60 min. (d) NIR-PAM signal intensity was detected at 1–60 min. (e) Signal change between signal before and after injection FINPs. (f) NIR-PAM *en face* images were acquired with energy of 180 nJ before injecting FINPs and with energy of 90 nJ after injecting FINPs. (g) SNR comparison of NIR-PAM images of different energy.

In the experiment, 0.7 ml of FINPs (1 mg/mL) were intravenously injected into rats via the tail vein. Then the rat fundus was time-serially monitored: 1 min, 3 min, 5 min, 15 min, 30 min, and 60 min after FINPs injection. The OCT *en face* image clearly shows the structure of the retina. And the intensity of OCT signal does not change significantly between 1 and 60 min after nanoparticle injection, shown in Figure 4A. In NIR-PAM *en face* image in Figure 4B, we can see small blood vessel like shadows between the major retinal blood vessels, and these shadows are absent in the OCT *en face* image.

For NIR-PAM image before FINPs injection, the fundus blood vessels on the side slight away from the needle ultrasound probe could not be distinguished at 0 min. The reason for the low SNR of the photoacoustic signal is that the hemoglobin excited by the pulsed laser could not produce higher photoacoustic signals. Compared with the PAM *en face* image at 0 min, a weak signal of the choroid vessels is visible at 1,064 nm wavelength in post-injection PAM *en face* images at 3 min. The enhancement of the photoacoustic signal by nanoparticles, as detected by an unfocused needle ultrasonic transducer, rapidly increases and eventually saturated, as illustrated in Figure 4B. After FINPs injection, specific vessel information can be easily visualized in NIR-PAM images. The reason is that the absorption coefficient of FINPs is high at the wavelength 1,064 nm. FINPs in blood vessels generate strong photoacoustic signals to clearly distinguish the location of detailed blood vessels at the RPE/choroid complex layer. In contrast, there is not a significant increase in PAM signal acquired at 1,064 nm without FINPs-injection. This phenomenon can remain 30 min after the injection of nanoparticles which provide an imaging scheme with higher speed and lower laser dosage.

As shown in Figure 4C and D, PAM signal and OCT signal reach peak in rat fundus from 1 min to 3 min after FINPs injection. The amplitude of PAM signal enhances from 0.267 (a.u.) at pre-injection to 0.572 (a.u.) at 1 min after injection. And OCT signal increases from 0.434 (a.u.) at pre-injection to 0.47 (a.u.) at 1 min after injection. When the maximum signal value is compared with the image before material injection, we find that the SNR of OCT increases by 8.23 %, and the SNR of NIR-PAM increases by 134 %, as shown in Figure 4E. However, it can be clearly seen in the chart that both the reflection and absorption signals are lower than 0 min at the 60th minute. The main reason is that the pigmented eyeball in rats directly exposed to the air for a long time will produce cataracts, which prevent most of the light from passing through the anterior segment. Furthermore, a small quantity of nanoparticles spills over the fundus blood vessels leading to further enhancement of the photoacoustic signal in the RPE layer or choroidal layer. Regions of superimposed photoacoustic signals that exceed the signal detector threshold appear white. In Figure S3 images, the imaging contrast is affected by the size related properties of the contrast agents, leading to leakage of some nanomaterials in blood vessels [36], [37], [38]. This extravasation results in a nanoparticles accumulation within the RPE layer or choroidal layer, which effectively intensifies melanin absorption and increases resultant photoacoustic signal. Hence, the anticipated negative contrast at the locations of the smaller retinal and choroidal vessels is not observed. Nonetheless, this phenomenon does result in an improved overall signal-to-noise ratio in the images. Specifically, after the injection of nanoparticulate material, the closely following extravasation into the perivascular space results in the smaller retinal vessels continuing to be delineated by shadows due to the impediment of signal absorption by the RPE layer. Concurrently, the choroidal vessels maintain their contrast through the differential absorption of melanin and hemoglobin within the RPE and choroidal layers. To corroborate our findings with more robust evidences, we conducted validations using SD albino rats devoid of melanin. Figure S4 illustrates that the contrast agent is present not only within the vasculature but also permeates into indeterminate locations. In the following study, the extravasation of the material is to be eliminated by regulating the size and the shape of nanoparticles, and the fundus layering and blood vessel segmentation algorithms is to be used to extract clearer and detailed fundus vascular network.

With FINPs substantially increasing the scattering and absorption signals, NIR-PAM can be used for molecular imaging with low power laser, which can significantly reduce the potential phototoxicity and effectively improve the safety of fundus tissues. In Figure 4F, the significant enhancement of the fundus photoacoustic signal by NIR-PAM after the injection of the FINPs contrast agent is demonstrated. Vessels in the healthy BN rat fundus were imaged using a 180 nJ pulse energy at 1,064 nm. Without the labeling of FINPs, hemoglobin exhibits low absorption while the RPE layer shows strong melanin absorption at 1,064 nm, rendering blood vessels indiscernible. However, due to the strong absorption of FINPs at 1,064 nm, the vascular contrast can be enhanced through injection into the tail vein. Employing a 90 nJ pulse energy at 1,064 nm allows for the partial recovery of blood vessels, albeit not the capillaries. Despite the pulse energy being halved, an improvement in the PAM signal amplitude is observed (Figure 4G). Without compromising imaging quality, a proper increase in dosage within the safe concentration range can result in even higher quality images.

### 3.3 FINPs enhanced NIR-PAM and OCT imaging of fundus in CNV model rats

**Figure 5:**
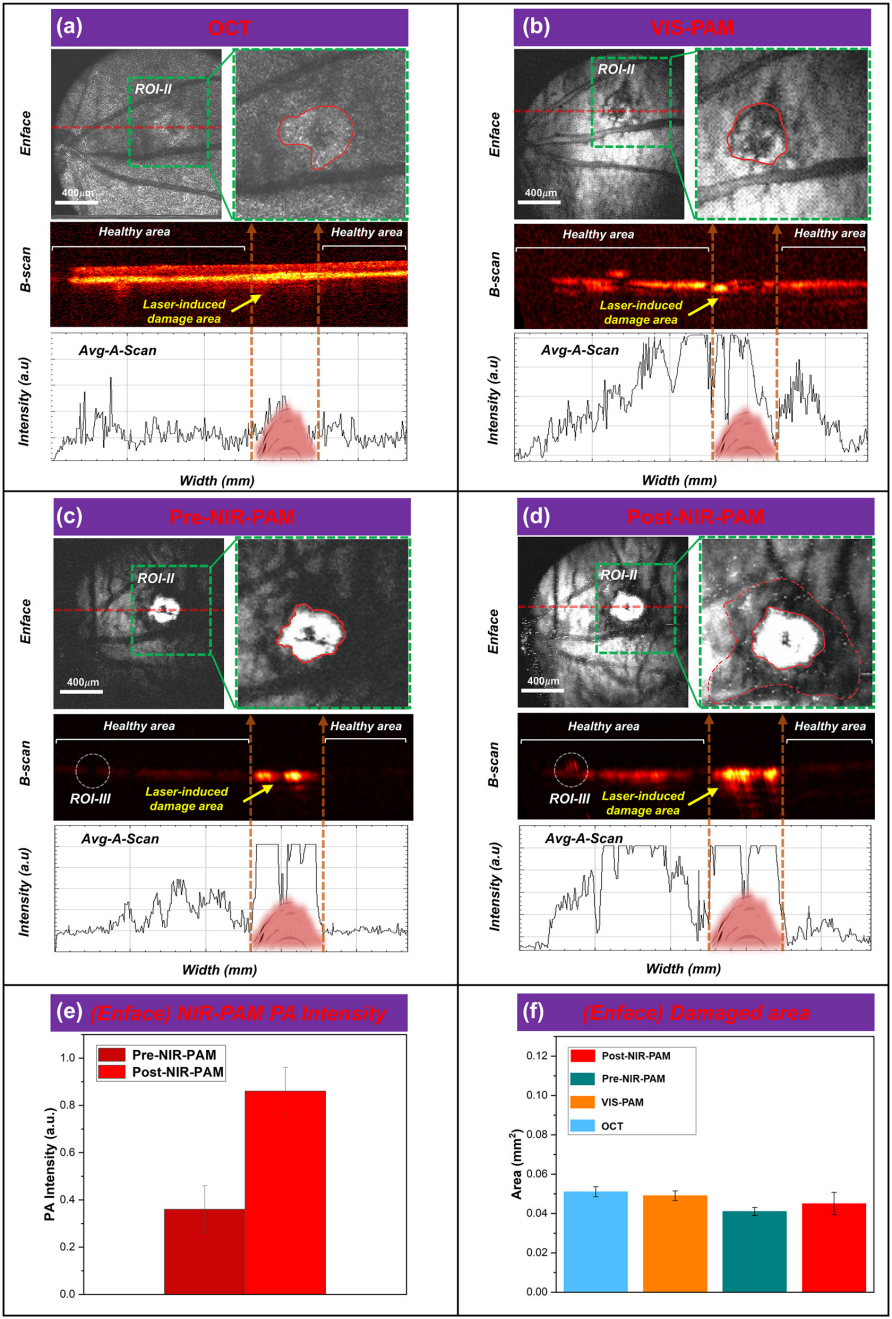
FINPs enhanced NIR-PAM and OCT imaging in CNV model rats. (a)–(d) OCT, VIS-PAM, pre-NIR-PAM, and post-NIR-PAM *en face* and B-scan image of rat fundus with nanoparticle injection. (e) The NIR-PAM signal amplitudes were enhanced 236.6 % from 0.363 (a.u.) to 0.859 (a.u.) at 3 min after injection. (f) ImageJ software to quantitatively analyze the laser-induced area.

The feasibility of imaging the complex fundus vascular activities of the CNV model rats (256 × 256 pixels) using VIS/NIR-PAM-OCT was subsequently verified, as illustrated in Figure 5A–D. As shown in Figure 5, discrepancies between the normal rat fundus and the CNV model rats fundus can be obviously differentiated. The area in the green dotted box is selected as the ROI-II (64 × 64 pixels) for further accurately quantitative calculation, and the red dotted line (II-Location) corresponds to one of the B-scan in the vascular structure *en face* mapping. The red irregular circle represents the lesion area in the enlarged image of the region of interest-II. For CNV model rat fundus VIS/NIR-PAM-OCT images, we adjusted the angle between the rat’s eyeball and the imaging beam along the *y*-axis by 30° *y*-axis to better display the details of the lesion area.

**Figure 6:**
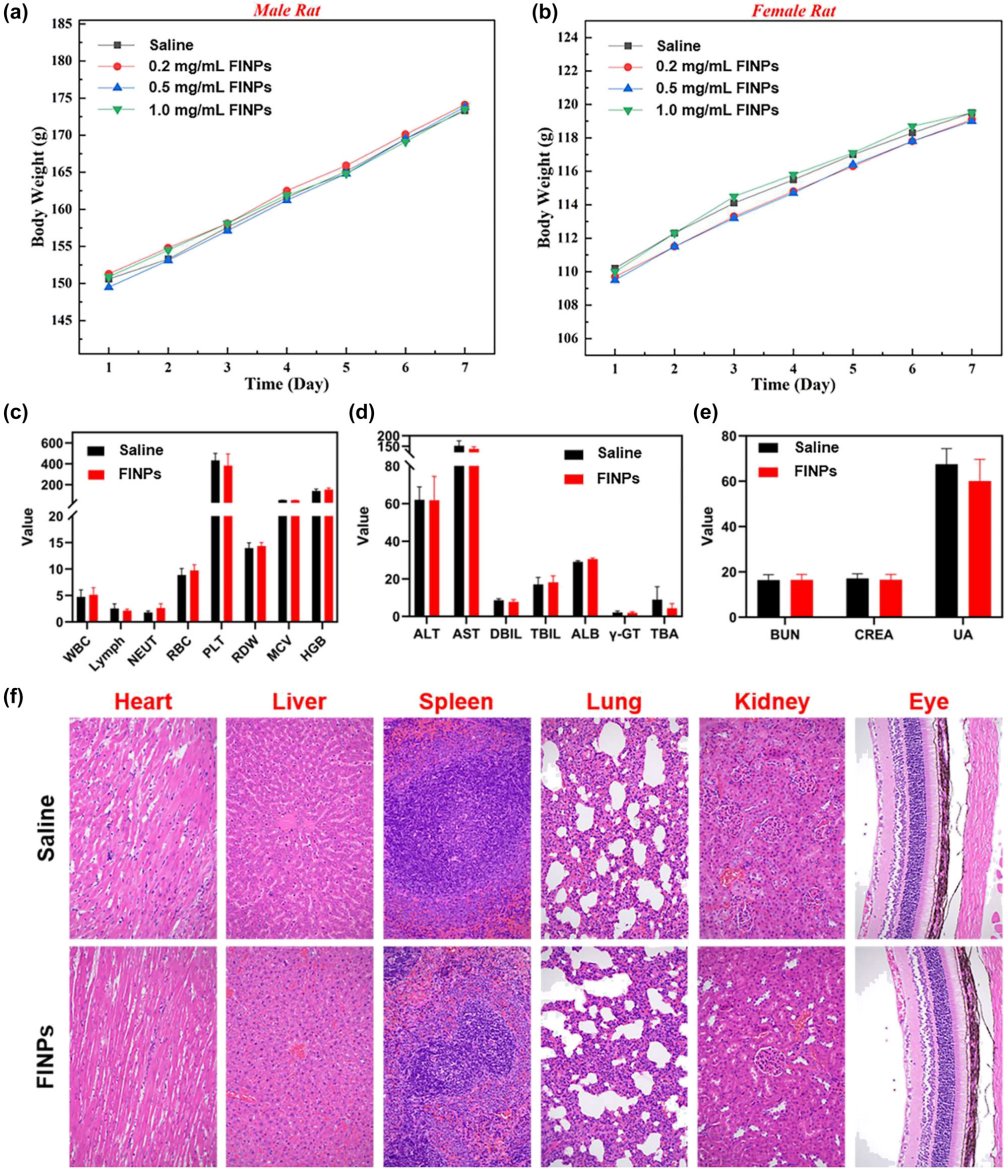
*In Vivo* toxicity assay and histological analysis after FINPs injection in rats. (a) Male rats injected with FINPs at different concentrations and saline during the 7-day experimental period. (b) Female rats injected with FINPs at different concentrations and saline during the 7-day experimental period. (c)–(e) Blood analysis of rats 7 days after injection of FINPs and saline. (f) Representative H&E stained images of major organs collected from rats sacrificed at 7 days after injection of FINPs and saline (magnification: 200×).

In Figure 5A, the lesion area is characterized by oval patches of hyperreflectivity in a globular pattern on OCT *en face* image (green dashed box) at 5 days after modeling. The lesion area is grayish white and CNV areas cannot be clearly distinguished. In the OCT B-scan image, the choriocapillaris hyperreflective subretinal lesion is easily differentiated from the surrounding retina and subretinal hyporeflective areas. An irregular, multi-layered, highly reflective area that protrudes into the subretinal space above the disruption of the highly reflective RPE layer appears in the OCT cross sectional image. Notably, OCT signals caused by the vessels are lower than circumjacent tissues. Subsequently, a signal plot was extracted from the OCT B-scan, clearly demonstrating a match between the intensity of the blood vessel in the OCT signals and the B-scan. As illustrated in Figure 5B, it can be seen that the hemoglobin in the blood vessels and the melanin in the RPE cells are strong absorbers of the illuminating light. This difference in optical absorption provides the foundation of functional and anatomical imaging of the blood vessels and the RPE. Detail distribution of new vessels over the laser photocoagulation region is shown in the VIS-PAM *en face* image, and the VIS-PAM B-scan image shows the neovascular growth in the RPE/choroid complex layer. However, the RPE/choroid complex layer vessels information cannot be well observed due to the absorption of visible light by hemoglobin and melanin. The average signal value of VIS-PAM related to the B-scan indicates that the gradient of pixel intensity on both sides of the laser-induced damage region is not apparent. In Figure 5C, signal of the lesion area in NIR-PAM *en face* image is saturated and many shadows around the white area of PAM image can be observed. Retinal blood vessels are observed as black shadows overlying the retinal RPE layer in NIR-PAM B-scan images due to the higher light absorption of melanin at about 1,064 nm compared to hemoglobin. The shadow of the lesion area is believed to be laser-induced rupture of Bruch’s membrane, and aggregated CNV provide the same strength of PAM signal. It is worth mentioning that although the overall pre-injection NIR-PAM (Pre-NIR-PAM) images SNR is lower, the signal in the laser-induced damage area is very strong, which are significantly different from the surrounding tissue signals. The *en face* and corresponding B-scan images can be seen that the laser-induced damage area signal strength is above the maximum value. In Figure 5D, due to the deeper penetration depth of near-infrared wavelengths, the image SNR is enhanced after injection of FINPs. The severity of CNV can be easily diagnosed from the Post-NIR-PAM images through the white area after laser-induced and the surrounding area of accumulated neovascularization. The white area (Red solid line irregular area in ROI-II in Figure 5C and D) shown in the images represents the region of laser induced CNV, which is typically indicative of the extent of tissue damage and subsequent inflammatory response. Surrounding this area (The red dotted irregular area in ROI-II in Figure 5D), the accumulated neovascularization can be discerned as a distinct perimeter or network of fine vessels, which are a hallmark of CNV progression. The contrast of the laser-induced zone and the neighboring regions provides a clear demarcation, thereby facilitating an accurate assessment of CNV severity. The post-NIR-PAM signal amplitudes enhance 236.6 % from 0.363 (a.u.) at pre-injection to 0.859 (a.u.) at 3 min after injection as shown Figure 5E. Changes of photoacoustic signal intensity in Figure 5C and D was quantitatively analyzed, which encompassed both signal within the CNV lesion area (indicated by the irregular red solid region) and that across the entire ROI-II (outlined by the green dashed box). To assess the extent of tissue damage, the dimensions of the highlighted region within the irregular red solid line circle were measured. Additionally, the process of CNV development within the green dotted line box (excluding the damaged area) was further analyzed.

We admit that signals saturated in the CNV area cannot be used for comparison. The PAM signals in CNV area is easily to be saturated even when illuminated with low light energy. The main reason of easy saturation in CNV area is that the modeling method can cause RPE cells to die due to thermomechanical damage. This results in the exposure and aggregation of melanin within the cells at the site of laser-induced injury. Compared with the surrounding regions, the damaged area accumulates more melanin. The photoacoustic signal generated from the laser-damaged area (CNV area) significantly exceeds that of the normal tissue. Previous studies [39], [40] indicate that a single RPE cell may contain more than 100 melanosomes, and upon exposure to irradiation above a threshold, it is likely to form an equivalent number of bubbles within the cell. The coalescence of bubbles around individual melanosomes into larger ones can result in death of RPE cells. Consequently, excessive stretching of the cell membrane can lead to membrane rupture or cell death, and the aggregation of melanin not enclosed by the cell membrane can produce a significant photoacoustic signal. This results in strong absorption even under 1,064 nm laser illumination without the addition of contrast agents.

To evaluate the NIR-PAM images can distinguish lesion areas, the *en face* images corresponding to the laser-induced area were analyzed in Figure 5F, respectively. A quantitative analysis of the laser-induced area was performed using ImageJ software, and it was found that there were no significant differences in the CNV area among the four *en face* images. Compared with VIS-PAM images, NIR-PAM can quantify the lesion area more easily. Compared with Pre-NIR-PAM images, after the injection of FINPs to improve the SNR, the RPE/choroidal composite layer blood vessel information can be observed. Although it is not easy to distinguish laser-induced damage regions area in OCT *en face* images, B-scan images information can be used as a good supplement, and OCT imaging can be used as imaging navigation in real time. In a nutshell, these high-quality results demonstrate that the VIS/NIR-PAM-OCT can detect the complicated vascular networks of fundus after FINPs injection.

### 3.4 *In Vivo* toxicity of FINPs

To further evaluate the health status of the rats and the toxicity of nanoparticles *in vivo*. The changes in body weight were monitored daily for seven days after the intravenous injection of nanoparticles. And hematological analysis and histopathological testing were performed as reliable indicators.

In this study, eight health BN rats with similar body weights (male: 150 g ± 1 g; female: 110 g ± 1 g) were selected. They were randomly divided into four groups each with two rats (one male, one female). Three groups of experimental rats were intravenously injected with FINPs at different concentrations (0.2 mg/mL, 0.5 mg/mL, and 1 mg/mL), while rats in the control group received intravenous injections of saline. As shown in Figure 6A and B, the time-serial body weight of rat injected FINPs at various doses are presented. On the seventh day, the rats both in experimental group and control group have similar body weights. Comparing the body weights of male rats and female rats injected with different concentrations of FINPs and saline, it can be observed that the body weight of rats is not impacted by nanoparticles. In the experiments, blood samples were collected from orbital venous on the seventh day of nanoparticle injection in rats at a concentration of 1 mg/mL of FINPs. Fasting was performed for 12 h before blood collection and rats were deeply anesthetized during the whole blood collection. Approximate 5 ml of whole blood obtained from experimental group and control group were dispensed into tubes containing anticoagulants. Then, samples were centrifuged at 3,000 rpm for 10 min to separate the serum from whole blood. All serum was stored at −20 °C until serological assays were carried out. There was no significant difference in hematological parameters between the experimental group and the control group (Figure 6C–E). Finally, all rats were sacrificed by cervical dislocation under deep anesthesia and a laparotomy was performed. Major organs of each animal were removed (Heart, liver, spleen, lung, kidney, and eye). About 1 cm^2^ of tissue was immersed in 10 % buffered formaldehyde for 24 h at room temperature. Subsequently, tissue samples were dehydrated in a dehydrator and embedded in paraffin. Tissue samples sections were cut at a thickness of 5 μm and then stained with hematoxylin and eosin (H&E). Images were obtained using a light microscope. Pathological examination of the main organs of the rats in the experimental group shows no inflammation (Figure 6F).

## 4 Discussion and conclusion

In this study, a multimodal VIS/NIR-PAM-OCT system was built to investigate the complementary information of optical absorption and scattering property of fundus, especially that of deep tissues. To enhance light absorption in the NIR band, FINPs was synthesized and investigated in NIR-PAM and OCT imaging. Results show that both NIR-PAM and OCT signals are enhanced effectively through intravenous injection of nanoparticles. For FINPs enhanced multimodal NIR-PAM and OCT images, OCT B-scan image can clearly show choroidal vessels under the RPE layer and NIR-PAM B-scan images show shadows at the RPE/choroid complex layer vessels. For CNV model rats, FINPs enhanced multimodal NIR-PAM and OCT imaging system can clearly and easily quantify the lesion area.

When comparing VIS-PAM and NIR-PAM imaging, it is observed that VIS-PAM is unable to reveal vessels within the RPE/choroid complex layer due to the penetration depth of the detection light. In FINPs enhanced NIR-PAM *en face* images, deep blood vessels can be visualized. The NIR-PAM images can be used to accurately quantify the fundus lesion area in the fundus of CNV model rats. More importantly, since the near-infrared light is much safer and more comfortable for ocular imaging, it is much suitable for fundus imaging.

Given that FINPs exhibit good biocompatibility and low toxicity alongside significant improvements in scattering and absorption properties, signal saturation was observed in the NIR-PAM image when utilizing the typical energy of 450 nJ at a 1,064 nm wavelength. This indicates the potential for reducing the intensity of incident light in NIR-PAM imaging. To the best of our knowledge, no study has been proposed to resolve this challenge. It is believed that by introducing a contrast agent can enhance imaging speed and lead to high-quality images by reducing the laser energy. Specifically, the employment of low-energy pulse signals is considered to improve the safety of eye imaging.

In this multimodal system, a typical SD-OCT system at the central wavelength of 840 nm was used, which clearly showed the cross sectional and *en face* images of the fundus. We admit that if an OCT system at central wavelength of 1 μm is used, the signal intensity will be significantly increased for the strong scattering of FINPs at NIR-II band, and the imaging depth of OCT will be also increased. Limited by the pulse repetition rate of the near-infrared semiconductor lasers for PAM imaging, only 5 kHz multimodal imaging was performed which could not be used to calculate the vascular distribution using optical coherence angiography (OCTA). However, compared with OCTA, PAM technique has the advantage of providing authentic vascular images with unequivocal molecule specific contrast and showing clear boundaries of ocular diseases such as CNV and tumors.

In the future work, NIR-PAM imaging of choroid will be further optimized. Nanoparticles with proper size and shape for strong light absorption and less material extravasation will be used to improve the SNR of PAM images. Retrobulbar injection of nanoparticulate material for *in situ* enhancement will be employed to improve the nanoparticle absorption in choroidal vessels [41]. Ultrasonic transducers with high center frequency (60 MHz–120 MHz) will be used to increase the longitudinal resolution of PAM for high depth discrimination [42], [43]. The development of algorithms for segmentation of the curved vessels will be a feasible scheme to facilitate the distinguishment of deeper vasculature.

In summary, a multimodal VIS/NIR-PAM-OCT imaging system was proposed to obtain structural and vascular information of the fundus. VIS-PAM images provided specific features of retinal vessles, and FINPs enhanced NIR-PAM and OCT could be used to accurately quantify lesions even in deep tissues of the fundus. This imaging technique of VIS/NIR-PAM-OCT may provide a promising tool for the diagnosis of fundus diseases in the future.

## Supplementary Material

Supplementary Material Details
