# Supplementary material for: Contrast-enhanced near-infrared photoacoustic microscopy and optical coherence tomography imaging of rat fundus
Source: Nanophotonics. 2024 Jun 13;13(19):3631–46. doi: 10.1515/nanoph-2023-0872 (PMC11465996; doi:10.1515/nanoph-2023-0872)
Supplement: Supplementary file 1 — Supplementary Material Details [file j_nanoph-2023-0872_suppl_001.pdf]

Supplementary Material for: Contrast-Enhanced Near-infrared Photoacoustic Microscopy and Optical Coherence Tomography Imaging of Rat Fundus

Fengxian Du[#1], Chen Niu[#1], Silue Zeng[#2], Jingqin Chen*[2], Chengbo Liu*[2], Cuixia Dai *[1]

[1] Shanghai institute of technology, Shanghai 201418, China

[2] Shenzhen institute of advance technology Chinese academy of sciences, Shenzhen 518055, China

*Corresponding author: jq.chen@siat.ac.cn, cb.liu@siat.ac.cn, sdadai7412@163.com

This supplemental material contains three Figures.

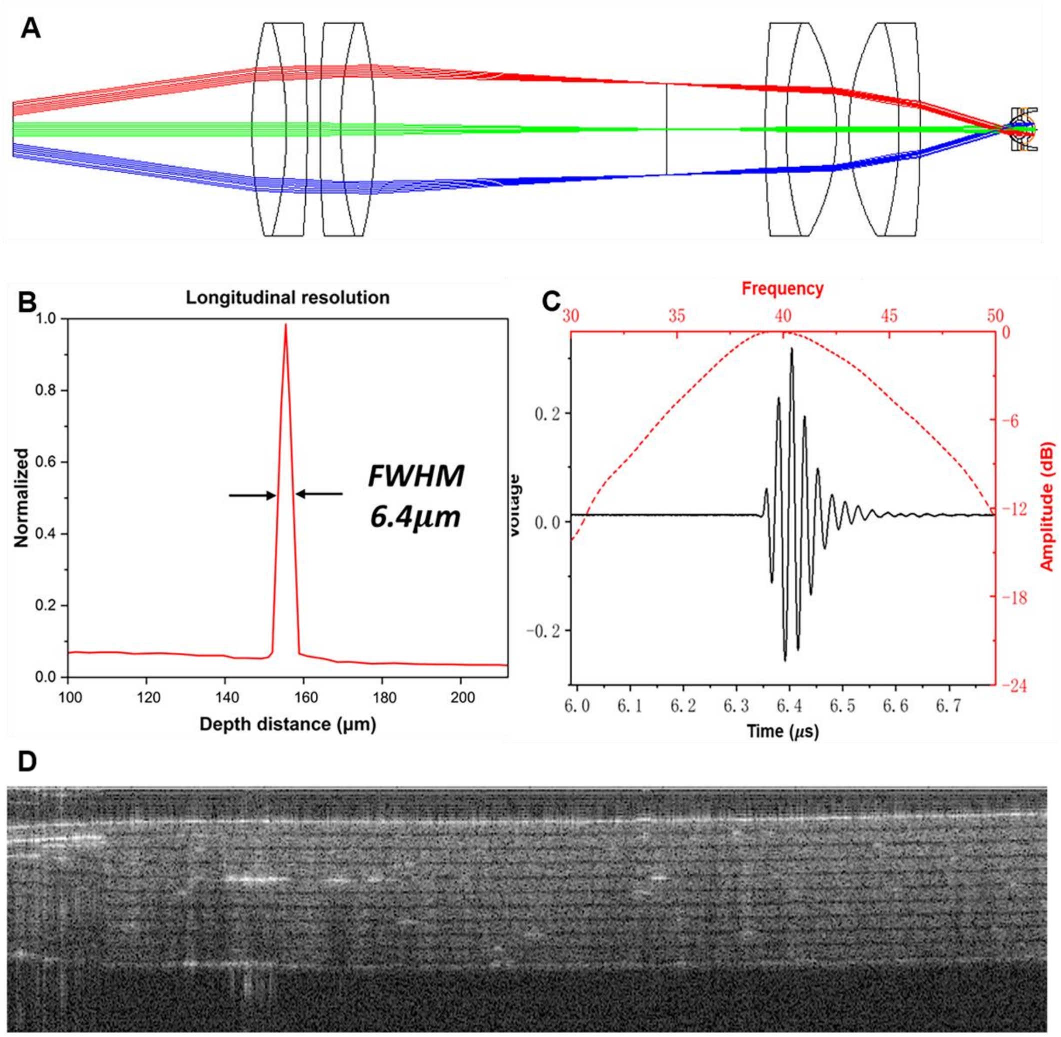

Figure S1. Imaging performance of the VIS/NIR-PAM-OCT. (A) ZEMAX optical path simulation of rat fundus. (B) Longitudinal resolution test of OCT system. (C) Performance of the unfocused ultrasonic transducer. (D) Depth-wise cross-sectional OCT B scan image of a Scotch tape section.

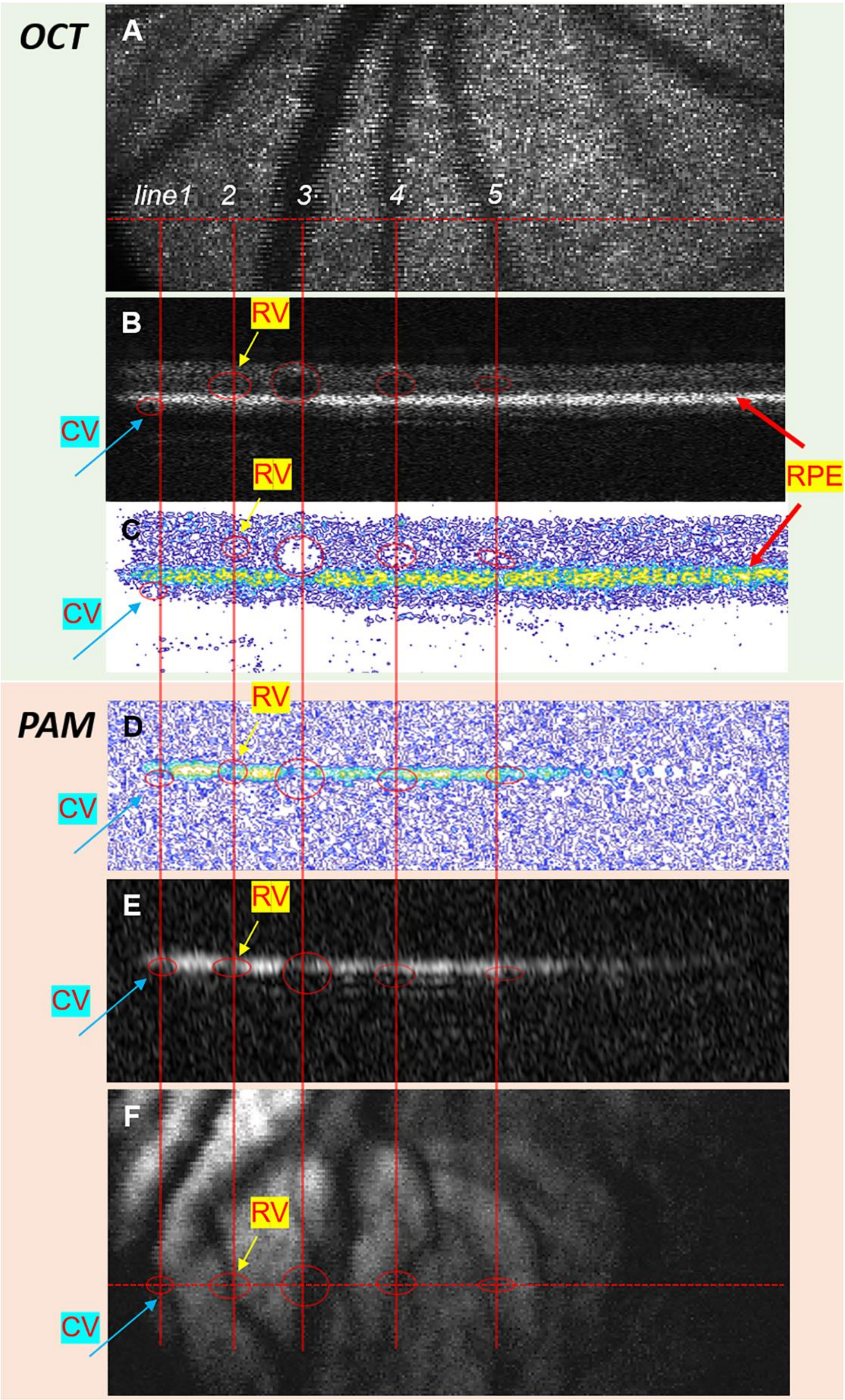

Figure S2. Multimodal PAM and OCT imaging of rat fundus. (A)-(C) OCT *en face*, B-scan and contour mapping image of rat fundus without nanoparticle injection. (D)-(F) NIR-PAM contour mapping, B-scan and *en face* image of rat fundus without nanoparticle injection. The dotted red line indicates the corresponding B-scan image. The red ellipse in the B-scan image indicates the location of blood vessels at the position of *en face* image line 1 – 5.

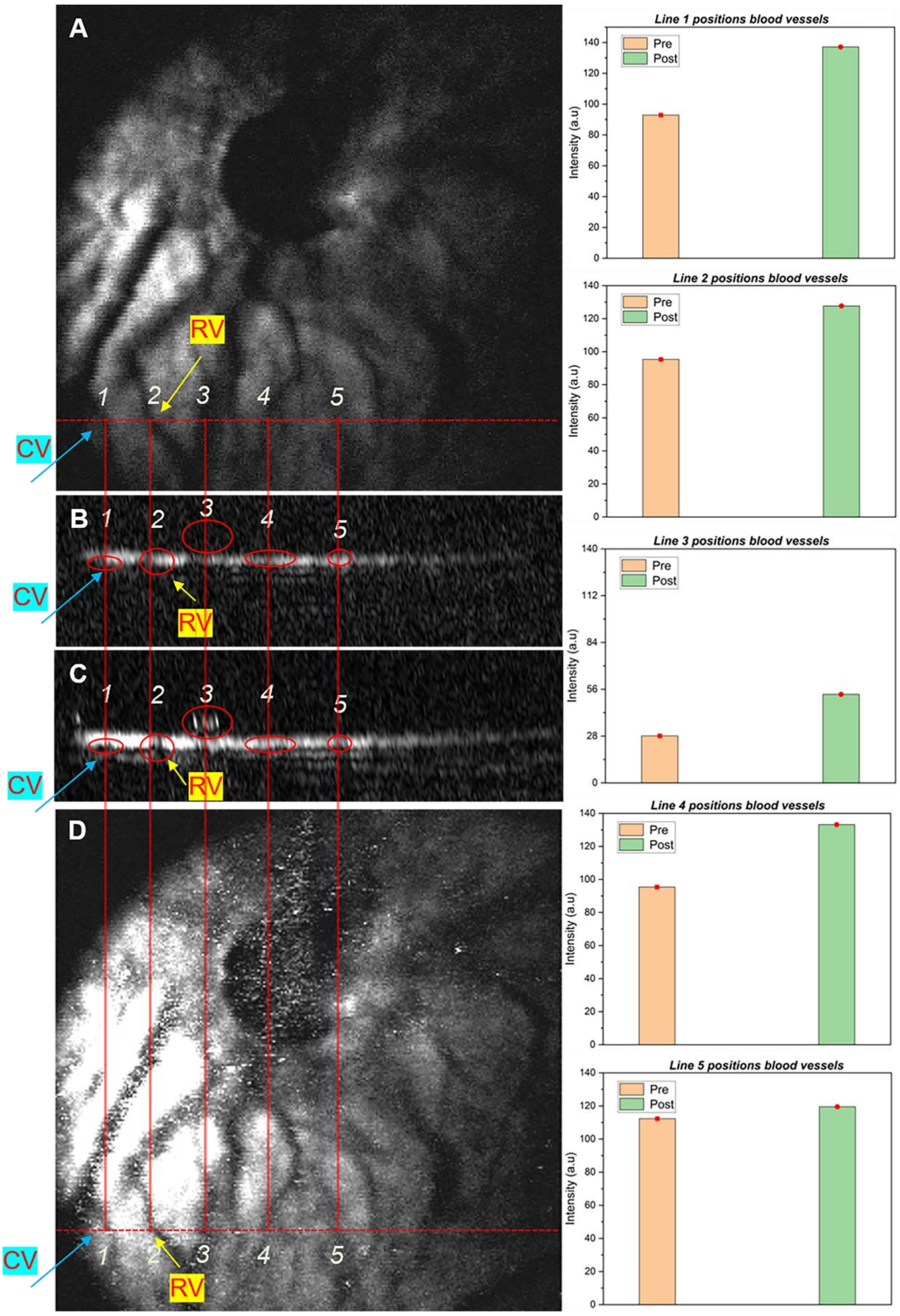

Figure S3. NIR-PAM imaging of rat fundus and changes in the intensity of injected nanoparticle at line 1-5. (A)-(B) NIR-PAM *en face* and B-scan image of rat fundus without nanoparticle injection. The dotted yellow line indicates the corresponding B-scan image. (C)-(D) NIR-PAM B-scan and *en face* and image of rat fundus with nanoparticle injection. The dotted red line indicates the corresponding B-scan image. The red ellipse in the B-scan image indicates the location of blood vessels at the position of *en face* image line 1 – 5.

In Figure S3 images, there is an obvious enhancement in the signal of the retina's larger blood vessels after the contrast agent application. In the case of smaller retinal and choroidal vessels, nanoparticles also improve the visualization of the detailed vascular network. To corroborate our findings with more robust evidences, we conducted validations using SD albino rats devoid of melanin. Figure S4 illustrates that the contrast agent is present not only within the vasculature but also permeates into indeterminate locations. In the following study, we plan to reduce the extravasation of the material by regulating the size of the material, and combine the fundus layering and blood vessel segmentation algorithms to further extract clearer and detailed fundus vascular network.

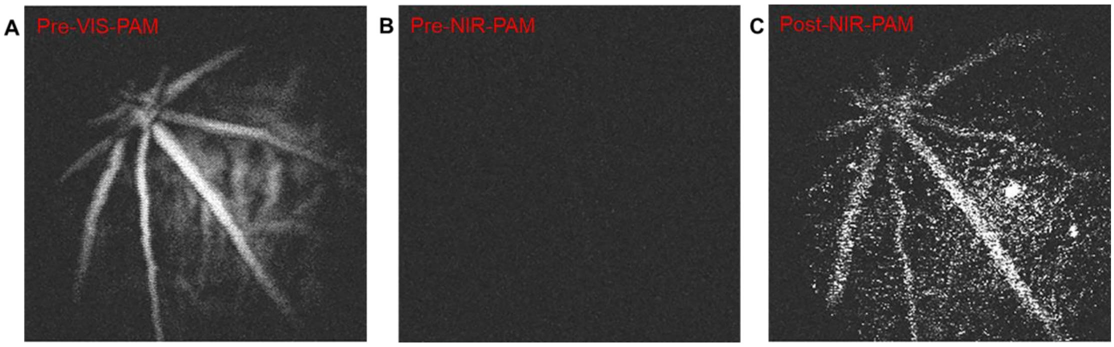

Figure S4. VIS/NIR-PAM imaging of SD rat fundus with nanoparticle injection.
